# Rethinking clinical oncology drug research in an era of value‐based cancer care: A role for chemotherapy pathways

**DOI:** 10.1002/cam4.3193

**Published:** 2020-06-10

**Authors:** J. Russell Hoverman

**Affiliations:** ^1^ Value‐based Programs US Oncology/McKesson Specialty Health Texas Oncology Dallas TX USA; ^2^ US Oncology/McKesson Specialty Health The Woodlands TX USA

**Keywords:** cancer management, chemotherapy, clinical cancer research, medical oncology

## Abstract

The United States spends nearly 1/5th of its GDP on healthcare. Yet, to achieve value‐based care, the Economist describes the US healthcare system as handicapped by multiple, disparate silos that prevent the organization and sharing of data. This paper explores the current state of clinical oncology drug research and its relationship to value‐based cancer care. Clinical Chemotherapy Pathways are proposed as a unifying structure to bring together disparate sources of data to increase value.

## Graphical abstract

There are many inconsistencies to cancer drug research that make it challenging to assign value. The systematic use of clinical pathways can enable the efficient use of novel therapies.

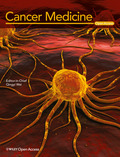

## 1 INTRODUCTION

In 2018, the United States spent 17.7% of GDP on healthcare. The per capita cost was $11172. Household spending is 28% of all funding sources.^1^ From May 2018 to May 2019 , the FDA approved 58 new drugs or new indications for the treatment of solid tumors and hematologic malignancies.^2^ All of these new drugs will be priced at substantial costs. A recent study by The Economist^3^ argued that the US healthcare system is handicapped in a value‐based environment by a myriad of disparate, and uncommunicative health information systems. This siloed system prevents various disciplines, groups, and institutions from organizing and sharing data. The current clinical cancer research component is among these.

To further characterize the relationships between novel drug research, cancer management, and Value‐based cancer care (VBCC), realizing that each may consist of multiple silos, this paper explores the salient features of each and considers the structure imposed by chemotherapy pathways in fostering collaboration.

## 2 ISSUES ABOUT VALUE IN CLINICAL ONCOLOGY DRUG RESEARCH

In a herculean effort, Hirsch and colleagues reviewed all interventional oncology studies from 2007 to 2010.^4^ Out of 40 790 studies, 8942 focused on medical oncology. About 62.3% were single armed, and 63.9% were non‐randomized. About 83% were phase 1 or 2; the average size was 51 patients. About 41.8% were funded by industry. The authors also noted “we identified more than 25 000 outcomes across oncology trials that occurred only once or twice.”

Additionally, Booth, et al showed that in the last three decades, industry sponsored trials have increased from 4% to 57% of total trials. Industry sponsorship was associated with a higher rate of endorsement of the experimental agent.^5^ The same group showed there was discordance between abstract presentations and published papers 63% of the time, 10% substantial.^6^ Chan et al reported that positive phase 2 trials led to positive phase 3 trials only 50% of the time.^7^ Industry sponsored trials were positive 89.5% vs 45% for all others. In 2009 Mathieu, et al^8^ reported that 45% of randomized clinical trials were registered with ClinicalTrials.gov. Of these, 35% had discrepancies between registered intended outcomes and outcomes published. About 83% of these incorporated statistically favorable results. Requirements for registration and identification of the primary endpoint (PEP) of the study have since become more stringent. More recent reviews indicate publication in abstracts of randomized trials more frequently “reported positive unplanned endpoints and unplanned analyses than negative outcomes in abstracts…”.^9^ Another review showed that of 134 registered studies with a clearly defined PEP, 14% published a PEP differing from that in the registry, 15% had issues with methodology, and 22% had problems with interpretation.^10^

There are additional concerns about the approval process of new drugs that are not well studied. (a) We know little about the difference in efficacy between drugs that are dosed slightly above the threshold response level and those dosed slightly below maximum tolerated dose. This is especially true for biologics and immune‐oncology (IO) drugs where there is high dose tolerance in a wide effective range.^11^ A recent report of dose intensity in Phase 1 drug trial showed responses in a wide range for IO drugs. For those with molecular or antibody targets, there was a general correlation of dose with response but stable disease was associated with a wide range of dosing.^12^ Even with cytotoxic drugs, while there is usually a tight correlation with dose and response, some drugs have doses reduced due to excess toxicity at the proposed dose.^13^, ^14^ (b) Once approved, fixed doses are recommended for some of the newer IO drugs, whereas the pivotal trials used weight‐based dosing. Although, vial size can make this challenging, being allowed to choose between dosing schemes would lower the overall cost.^15^ (c) Some studies use vastly more expensive drugs in combinations when lower cost drugs are available. Gemcitabine/nab‐paclitaxel in pancreas cancer is an example.^16^ Recent trials of IO drugs with nab‐paclitaxel are also relevant.^17^ (d) Some studies have more than one intervention, making outcomes and value decisions difficult to isolate. Examples are those studies with an induction phase and a maintenance phase. In the maintenance phase, a drug is given for maintenance without a control arm or a drug with unknown benefit is piggybacked onto a drug considered standard of care. As an example, in lung cancer, bevacizumab was continued as maintenance with no standard treatment control arm.^18^ The Point Break study^19^ is an example of the latter, where the two maintenance arms were bevacizumab alone or in combination with pemetrexed. Neither was compared with the standard of care, which would have been pemetrexed alone. Bevacizumab has also been piggybacked with capecitabine without a capecitabine alone control arm.^20^ The pivotal trial of pemetrexed‐carboplatin with or without pembrolizumab has a maintenance arm following the three‐drug combination of pembrolizumab for 24 months along with pemetrexed indefinitely. The control arm with standard chemotherapy has pemetrexed maintenance only. As this is a novel combination, there is no known harm or benefit to maintenance of any type, yet no placebo or start/stop strategy or pembrolizumab alone or pemetrexed alone arm was studied as an option for maintenance with the combination therapy. With these drugs in combination, there is an enormous monthly cost without measured value.^21^ (e) In a study by Hilal, Sonbol and Prasad, 97 studies that were tied to approval of 95 new cancer drugs were evaluated for the appropriateness of the control arm, that is whether the control arm represented optimal standard of care. Of these randomized controlled trials, 17% had suboptimal control arms.^22^ (f) Randomized trials may have uncertain applicability to the usual medical oncology population of patients. Those patients on research trials are younger and healthier with fewer medications and comorbidities.^23^, ^24^ (g) Some studies may be marketed inappropriately when considering actual practice in a cost‐effective environment. The media marketing of pegfilgrastim (gcsf) in metastatic breast cancer is based on a study of docetaxel given at a dose of 100 mg/M^2^ with or without gcsf.^25^ Docetaxel at that dose as a single agent is now rarely used. Another study showed there was no survival difference among doses of 100 mg/M^2^, 75 mg/M^2^, and 60 mg/M^2^.^26^ Less intense dosing is consistent with American Society of Clinical Oncology guidelines for treatment of solid tumors in the non‐curative setting.^27^ (h) Study PEPs may not translate into meaningful survival differences.^28^ Two recent studies with bevacizumab in ovarian cancer highlight the discordance between progression free survival (or response rate) and overall survival.^30^, ^31^ (i) Recent reports indicate that drugs given accelerated approval do not always translate the initial promise into meaningful survival benefit. Gyawali et al^31^ showed that of 93 drug indications given accelerated approval, 20% showed improvement in survival, 20% showed improvement in the same surrogate measures as in the initial study, and 21% showed improvement in different surrogate measures. The remaining studies were ongoing, pending, or delayed. Kim and Prasad^32^ found similar results: 57% of 54 drugs approved have “unknown affects on overall survival or fail to show gains in survival.” Particularly notable is the case of bevacizumab in the treatment of glioblastoma. An initial improvement in response rate was not proven to lead to improvement in survival in the confirmatory trial. The bevacizumab cohort also had increased toxicity. Yet, bevacizumab received full approval for this disease.^33^

To summarize current oncology research: Published trials have widely varying structures and outcomes. There are fewer impartially funded trials. Reported outcomes may be skewed toward statistically positive findings. Drug choice in combination trials may be done without consideration of lower cost alternatives. Drug choice in maintenance therapies may be made without clinical evidence. Marketing of trial results may not be associated with real world use and may even be counter to generally recommended guidelines. Surrogate endpoints may lead to drug acceptance or approval without improvement in meaningful outcomes. There is, as Hirsch et al^4^ mention, the “lack of a standard ontology” that would allow comparisons across trials and even across databases.

These considerations do not address directly the issue of “multiplicity” raised by Prasad and Booth.^34^ Multiplicity becomes a concern when there are “many trials testing similar hypotheses with similar drugs (such that) the likelihood that any one trial will yield a significant result is increased by the large number of times that something has been tested.” The analysis here outlines the structural difficulties with the conduct of clinical trials and subsequent marketing that make multiplicity possible.

## 3 VALUE‐BASED CANCER CARE

The Economist report defines value‐based care as “the creation and operation of a health system that explicitly prioritizes health outcomes that matter to patients relative to the costs of achieving those outcomes.” There are four domains for this enterprise: (a) An enabling structure; (b) Explicit measurement of outcomes and costs; (c) Integrated patient‐centered care; and (d) A payment system based on outcomes, not volume.^3^

Value‐based delivery models are rapidly inserting themselves into the cancer care delivery complex. This is particularly true for Medicare‐aged patients. Cancer is predominantly a disease of the elderly. Most oncology practices will have Medicare, either as traditional fee‐for‐service Medicare or as Medicare Advantage for over 50% of new cancer patients. For traditional Medicare, there is the Oncology Care Model (OCM), a value‐based program developed by the Center for Medicare and Medicaid Innovation (CMMI).^35^ For practices participating in the OCM, up to 20%‐30% of all new cancer patients will be covered by this program.

One goal of the OCM was to create a template for Medicare Advantage and commercial insurers to use. United Healthcare and Aetna have published results of earlier models.^37^, ^38^, ^39^ They, as well as Cigna and Humana have programs that are operational or in development. Anthem and some of the regional Blue Cross insurers have also implemented value‐based cancer programs. Although many of these programs are in the beginning stages, if effective, most oncology practices will have 50%‐70% of all new cancer patients covered by a value‐based delivery system within the next 3‐5 years. To emphasize, this means that, in the near future, the typical oncology practice will have over 50% of their patients for whom the total cost of care will be important––their reimbursement linked directly to how well they meet the requirements of the value‐based payment model. How well oncologists can manage the total cost of care will impact the financial health of these practices.

It is clear the current state of clinical oncology research is not designed to support the goals of VBCC. Trials are designed to measure outcomes that lead to FDA approval. Cost is not a consideration. In some trials, cost is added without any evidence of benefit or without studying less expensive alternative regimens. The trials do not typically assess the cancer patients we see in our clinics.

## 4 USING PATHWAYS AS AN ENABLING STRUCTURE

The incorporation of Pathways into these recommendations requires some explanation. The Pathways programs were initiated to address situations where multiple regimens had similar outcomes in specified clinical situations. One early example was in metastatic non‐small cell lung cancer where there were four equally effective regimens.^39^ The primary tenet of a Pathways program was to evaluate outcomes and toxicity first and if these were the same for two or more regimens, costs could be considered as a deciding factor. Subsequent studies have shown that using Pathways can reduce drug costs.^41^, ^42^, ^43^, ^44^, ^45^ The American Society of Clinical Oncology has developed recommendations for legitimizing Pathways programs for chemotherapy selection.^45^ Payers look favorably on Pathways programs and have developed products of their own.^46^ The principles of Pathways can be applied to other settings beyond clinical chemotherapy trials.^48^, ^49^ The key to these programs is meticulous assessment of the evidence for efficacy and toxicity and, only then, consider costs.

The challenge for oncology and medicine in general is to operationalize changes that enable alignment of clinical drug research with VBCC to improve outcomes and reduce costs. Enabling changes might include: (a) Develop an ontology and supporting structure across all research platforms. (b) Develop interoperability of electronic medical record (EMR) system platforms to get complete information on unstudied patient groups, such as those 70 and 80 years old, those on multiple medications and/or with comorbidities. This would allow practices and payers to use Real World Evidence (RWE) to answer questions about the impact of new drugs or other interventions on the outcomes and, therefore, value for these otherwise unstudied patients. (c) Standardize and measure patient reported outcomes, especially for the elderly. (d) Develop interoperability among EMR and claims databases, including Medicare, to measure total cost of care. RWE would bring together clinical information and claims data to study these critical cancer populations which represent the majority of patients in clinical practice. (e) Form contract relationships of Medicare and large payers with validated Pathways owners to use Pathways as a tool to assess the value of the research structure and outcomes. (f) Manufacturers would continue the current processes for FDA approval for new drugs. However, a new drug or regimen would have to demonstrate comparative value for a specific indication to be placed on Pathways. (g) If there were more than one option for a particular Pathway indication, an insurer, including Medicare, could make a coverage decision to narrow the choice based on value. (h) The specific Pathway indication could vary by age, comorbidity, and other risk factors. (i) Pathways could legitimize substitution of lower cost drugs known to be equivalent given with the same dosage and schedule, (j) Pathways could reject studies with surrogate endpoints, unmeasured variables, or inappropriate controls, (k) Pathways could do the follow‐up needed for drugs given expedited approval, and (l) Where there are more than one choice for a Pathways indication, payers, including Medicare could negotiate Pathways placement based on price.

## 5 PATHWAYS AND THE QUEST FOR VALUE MEASURES

Finding a consensus among practicing oncologists about value will be challenging. Yet “Defining a common understanding and measurement of value is a critical and necessary first step in improving the value of cancer care in the United States.”^49^ At the same time “Both quality and statistical precision have important implications for the creation and interpretation of value.”^50^

There are constructs available to assess the value of a cancer drug. The European Society of Medical Oncology (ESMO),^51^ the American Society of Clinical Oncology (ASCO),^52^ and the National Comprehensive Cancer Network (NCCN)^53^ among others have primarily patient‐oriented formats. However, none are placed in a structure to compare regimens by disease and line of therapy with payment implications in a value‐based environment.^51^, ^55^ The ASCO and ESMO calculations do not specifically include costs. In the NCCN evidence blocks, the costs determinations are not consistent.^53^ Improving on these formulations requires attention to evidence, addressing the concerns with clinical oncology drug research outlined here, and placing the assessments in an enabling structure tied to clinical decision‐making and reimbursement. A Pathways environment can do this.

It is an interesting exercise to consider what, for example, the ESMO and ASCO value frameworks would look like in a Pathways structure. As a Pathways program would have assessed these, there would be no points awarded for disease‐specific outcomes and patients would receive an accurate, validated representation of survival, either in a survival curve, or a bell‐shaped curve for survival or specific numbers for median, 6‐month, 1‐year, 2‐year, and 5‐year survival.^55^ This would be accompanied by cost numbers based on Medicare allowable per month and for treatment duration. Framework points would be awarded for various aspects of patient specific outcomes and cost. These could include projected inpatient and direct and indirect outpatient costs, toxicities, symptom burden with various patient reported concerns, incapacity, dependence, and other indicators meaningful for patients. This would allow for objective discussions of survival and costs and extensive exploration of patient values.

Nothing of this discussion suggests rationing of care, as the concern is that the current system does not adequately address the opportunity to reduce the use of low value care, and to negotiate pricing on value. The least controversial strategy to reduce costs and improve value is to eliminate instances of low or no value.

## CONFLICTS OF INTEREST

None to disclose.

## AUTHOR CONTRIBUTION

All aspects of the manuscript.

## Data Availability

Data sharing are not applicable as no new data were created of analyzed in this paper.
